# Life Cycle of the Cardiac Voltage-Gated Sodium Channel Na_V_1.5

**DOI:** 10.3389/fphys.2020.609733

**Published:** 2020-12-17

**Authors:** Caijuan Dong, Ya Wang, Aiqun Ma, Tingzhong Wang

**Affiliations:** ^1^Department of Cardiovascular Medicine, First Affiliated Hospital of Xi’an Jiaotong University, Xi’an, China; ^2^Key Laboratory of Molecular Cardiology, Shaanxi Province, Xi’an, China; ^3^Key Laboratory of Environment and Genes Related to Diseases, Xi’an Jiaotong University, Ministry of Education, Xi’an, China

**Keywords:** Na_V_1.5, *SCN5A*, biosynthesis, trafficking, anchoring, degradation, gating modulation, post-transcriptional modification

## Abstract

Cardiac voltage-gated sodium channel Na_V_1.5, encoded by *SCN5A*, is crucial for the upstroke of action potential and excitation of cardiomyocytes. Na_V_1.5 undergoes complex processes before it reaches the target membrane microdomains and performs normal functions. A variety of protein partners are needed to achieve the balance between *SCN5A* transcription and mRNA decay, endoplasmic reticulum retention and export, Golgi apparatus retention and export, selective anchoring and degradation, activation, and inactivation of sodium currents. Subtle alterations can impair Na_V_1.5 in terms of expression or function, eventually leading to Na_V_1.5-associated diseases such as lethal arrhythmias and cardiomyopathy.

## Introduction

**FIGURE 1:**
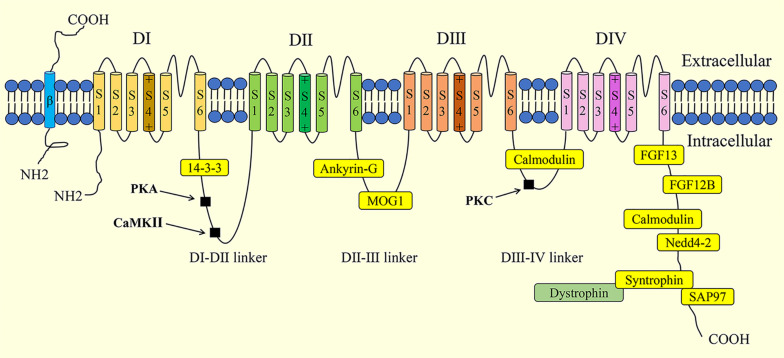
Schematic illustration of the structure of cardiac sodium channel (Na_V_,1.5) and its interaction proteins. Phosphorylation sites are indicated in black squares. PKA, protein kinase A; CaMKII, Ca^2+^/Calmodulin-dependent kinase II; PKC, protein kinase C; FGF, fibroblast growth factor; SAP97, synapse-associated protein 97.

The cardiac voltage-gated sodium channel Na_V_1.5 is made up of a 220-kDa pore-forming α-subunit and 30-kDa auxiliary β-subunits. For the convenience of reading, Na_V_1.5 in the following parts refers to the α-subunit of Na_V_1.5. The *SCN5A* gene, found on chromosome 3p21, encodes the Na_V_1.5 channel, which includes four homologous domains (DI-DIV), a C-terminus, and an N-terminus (Figure 1). Each domain of the Na_V_1.5 channel is composed of six membrane-spanning segments (S1-S6). Specifically, the S4 transmembrane segment acts as a voltage sensor. The S5 and S6 transmembrane segments form a pore with the intermembrane P-loop, which determines ion selectivity and influx (Jiang et al., 2020). The Na_V_1.5 channel mediates the rapid influx of sodium ions (Na^+^), which triggers cardiac action potential (AP), induces rapid depolarization, and initiates the excitation-contraction coupling cascades in the cardiomyocytes (Han et al., 2018). The sodium current (*I*_Na_) generated by Na_V_1.5 is determined by the channel density on the plasma membrane and channel gating. Na_V_1.5 is traditionally regarded as a monomer. However, it is well accepted that Na_V_1.5 is assembled as a dimer and exhibits coupled gating properties (Clatot et al., 2017). β1-β4 subunits, encoded by the SCN1b-4b genes, consist of an extracellular N-terminus, a transmembrane α-helix, and an intracellular C terminus (Salvage et al., 2020). The β-subunits act as gating modulators of Na_V_1.5 and facilitate Na_V_1.5 residence at the intercalated disk.

The life cycle of Na_V_1.5 begins with the transcription of the *SCN5A* gene and RNA processing. Then, Na_V_1.5 proteins are synthesized at the rough endoplasmic reticulum (ER) and follow the secretory pathway to reach the plasma membrane where the proteins exert their respective function. Various proteins are reported to be involved in the biosynthesis, trafficking, anchoring, post-transcriptional modification, gating regulation, and degradation of Na_V_1.5 by directly or indirectly interacting with the different motifs and domains of Na_V_1.5.

The aim of this review is to provide an updated account of the life cycle of Na_V_1.5, while summarizing *SCN5A*-related diseases and novel therapeutic strategies targeting Na_V_1.5.

## Biosynthesis

**FIGURE 2:**
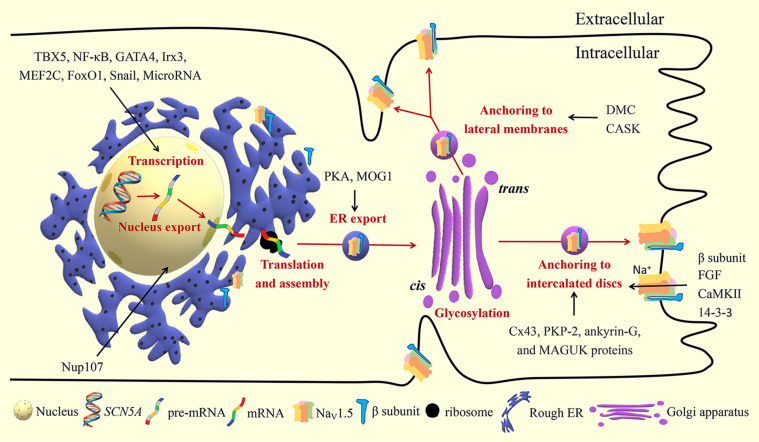
Biosynthesis and trafficking of Na_V_1.5. TBX5, T-box transcription factor 5; NF-κB, nuclear factor kappa-B; Irx3, Iroquois homeobox 3; MEF2C, myocyte enhancer factor-2C; Forkhead box O1; Nup107, nucleoporin 107; PKA, protein kinase A; Cx43, connexin 43; PKP-2, plakophilin 2; MAGUK, membrane-associated guanylate kinase; DMC, dystrophin-syntrophin multiprotein complex; CASK, calcium/calmodulin-dependent serine protein kinase; FGF, fibroblast growth factor; CaMKII, Ca^2 +^/ calmodulin-dependent kinase II.

Gene expression in eukaryotes starts with transcription, the transition of a DNA sequence into an RNA transcript (known as precursor mRNA) assisted with RNA polymerase and transcription factors. The precursor mRNA is the copying of the coding and noncoding genes which then need to undergo splicing and post-transcriptional modifications to produce mature mRNA and ultimately translate into polypeptides (Figure 2). The human *SCN5A* gene spans approximately 8,000 bp and consists of 28 exons, which can be spliced together to generate the full-length Na_V_1.5 protein (about 2016 amino acids). Exons 2–28 encode the protein-coding sequence. Exon 1 and part of exon 2 encode the mRNA 5′-untranslated region (5′-UTR). Exon 28 encodes the mRNA 3′-UTR (Wang et al., 1996). The transcription of the *SCN5A* gene is regulated by various transcription factors. Positive transcription factors such as TBX5 and NF-κB increase *SCN5A* mRNA abundance and further increase the expression and current of Na_V_1.5, while negative transcription factors such as FOXO1 and Snail show the opposite effects (Table 1). MicroRNAs (miRNAs) regulate gene expression through translational repression or mRNA degradation. miR-192-5p is reported to negatively regulate the expression of Na_V_1.5 and reduce *I*_Na_ density by interacting with the 3′-UTR of human *SCN5A* mRNA (Zhao et al., 2015).

**TABLE 1:** Transcription factors of Na_V_1.5 and their binding sites.

Transcription Factors		Binding Sites	Effects	References
Positive	TBX5	an enhancer downstream of the *SCN5A* gene	The knockout of TBX5 from the mature murine ventricular conduction system markedly decreases the density of Na_V_1.5, increases arrhythmias propensity, and results in sudden cardiac death.	Arnolds et al., 2012; Ivan et al., 2004; Steimle and Moskowitz, 2017
	NF-κB	the *SCN5A* promoter region	Treatment with angiotensin II (AngII) or H_2_O_2_ enhances the binding capacity of NF-κB with NF-κB binding sequence, thus significantly decreasing the *SCN5A* mRNA levels and *I*_Na_ in the H9C2 myocytes. Mutations in the NF-κB binding site at the *SCN5A* promoter region mitigates the decreased activity of Na_V_1.5 in response to AngII and H_2_O_2_.	Shang and Dudley, 2005; Shang et al., 2008
	GATA4/5	within the *SCN5A* proximal promoter and intron 1 regions	GATA4 and GATA5 bind to the GATA binding site within the *SCN5A* proximal promoter and intron 1 regions to synergistically activate the *SCN5A* gene in the human left ventricle samples. The GATA4^+/–^ heterozygous mice exhibited short PR intervals.	Brewer and Pizzey, 2006; Munshi et al., 2009; Schluterman et al., 2003; Tarradas et al., 2017
	IRX3		In the HL-1 cardiomyocytes and neonatal mouse ventricular myocytes, overexpression of IRX3 upregulated the mRNA levels of *SCN5A* and Cx40, which was not observed by overexpressing mutant IRX3. Deletion of IRX3 and IRX5 in the myocardium prolonged the atrioventricular conduction, which is partly due to the activation of Na_V_1.5 expression	Gaborit et al., 2012; Koizumi et al., 2016
	MEF2C	within the *SCN5A* promoter region	Hu antigen R (HuR) increased the mRNA expression of MEF2C by inhibiting MEF2C mRNA decay and consequently enhancing *SCN5A* mRNA transcription. Consistently, these effects were attenuated upon transfecting the cardiomyocytes with MEF2C siRNA.	Zhou et al., 2018a; Zhou et al., 2018b
Negative	FOXO1		FOXO1 negatively regulated Na_V_1.5 expression by binding to the *SCN5A* promoter and inhibiting its activity in the HL-1 cardiomyocytes. Deletion of FOXO1 increased the mRNA and protein levels of Na_V_1.5 and *I*_Na_ in the ventricular myocytes.	Cai et al., 2014; Mao et al., 2012
	Snail	E-box sites in the *SCN5A* core promoter	Overexpression of Snail in mice resulted in downregulated *SCN5A* mRNA level, reduced Na_V_1.5 level and decreased *I*_Na_. CNS28^–/–^ mice, which disrupted the Snail-binding region regulator, exhibited significantly upregulated SCN5A mRNA level, increased Na_V_1.5 level and enhanced *I*_Na_ in the freshly isolated ventricular myocytes.	Atack et al., 2011; Hesse et al., 2007; Nieto, 2002

Alternative splicing creates multiple variants of Na_V_1.5 including functional and nonfunctional types (Schroeter et al., 2010). Compared to full-length Na_V_1.5, spliced Na_V_1.5 variants like Na_V_1.5a, Na_V_1.5d, and Na_V_1.5e present altered electrophysiological kinetic properties; Na_V_1.5c show unchanged electrophysiological properties; Na_V_1.5b, Na_V_1.5f, and truncated variants E28B-E28D do not generate currents (Schroeter et al., 2010). The splicing machinery might explain the inconsistent genotype-phenotype outcomes. For instance, the LQT3-associated mutation *SCN5A*-T1620K showed electrophysiological properties similar to those of hNa_V_1.5, hNa_V_1.5a, and hNa_V_1.5c, but different effects in the hNa_V_1.5d background (Gaetano et al., 2011). Nevertheless, the exact mechanisms of Na_V_1.5 splicing regulation are still unclear but involve species-dependent, tissue-specific, and developmental factors.

Na_V_1.5 splice variants are reported to be involved in the pathophysiology of heart failure (HF), *SCN5A* channelopathies, and myotonic dystrophy. With angiotensin II (AngII) or hypoxia stimuli (signals common to HF), activated splice factors RBM25 and hLuc7A increase Na_V_1.5 splice variants E28C and E28D. Increased E28C and E28D variants cause a reading frame shift and encode truncated Na^+^ channels before DIV of the channel due to premature stop codons (Gao et al., 2011). The nonfunctional channels are trapped in the endoplasmic reticulum (ER) and trigger the unfolded protein response (UPR). UPR activation further leads to destabilization of the remaining full-length *SCN5A* mRNA, exacerbating the reduction of *I*_Na_ (Gao et al., 2013; Noyes et al., 2017). Studies also found that *SCN5A* splicing in cardiomyocytes and white blood cell (WBC) changes in a correlated manner during HF. If altered WBCs *SCN5A* variants were proven to increase the risk of sudden death or other HF-associated arrhythmias, a blood examination to predict HF-associated arrhythmic risk would be possible (Gao and Dudley, 2013).

## Trafficking and Anchoring

*SCN5A* mRNA exported from the nucleus is translated to protein and assembled in the rough ER. Next, the protein is translocated to the Golgi apparatus for further modification. After exiting the Golgi apparatus, Na_V_1.5 settles in the plasma membrane to exert its functions.

### Nucleus Export

Nuclear pore complexes, also known as nucleoporins, mediate the exchange of molecules between the nucleoplasm and cytoplasm. Nucleoporin 107 (Nup107) selectively facilitates the export of *SCN5A* mRNA from the nucleus to cytoplasm without affecting the *SCN5A* mRNA level (Guan et al., 2019). Additionally, increased Nup107 and Na_V_1.5 were observed in the cardiomyocytes and heart tissues under hypoxic and oxidative stress conditions. This suggests that Nup107 is a protein that reacts rapidly to the insults and can be developed as a novel molecular therapeutic target for myocardial ischemic damage.

As of writing, it is not clear whether there are other nucleoporins that take part in the nucleus-cytoplasmic trafficking of *SCN5A* mRNA. MOG1 is found to interact with Ran GTP, a small protein that mediates the nucleus import and export of proteins and RNA (Steggerda and Paschal, 2001). MOG1 is shown to promote intracellular trafficking of Na_V_1.5 from the ER (Yu et al., 2018). Thus, MOG1 may also play a regulatory role in the nucleus-cytoplasmic trafficking of *SCN5A* mRNA.

### ER Export

ER is the first station of the secretory pathway where the processes of protein folding, quality control, and protein complex assembly take place. Several ER retention and export motifs have been identified in ion channels (Mikosch and Homann, 2009; Benyair et al., 2011). ER export motifs are crucial for efficient ER export of mature and properly folded proteins through coat protein II (COPII) vesicles. COPII-coated vesicles in association with Sec23/24, Sec13/31, Sar1, and Sec12 mediate anterograde protein trafficking between the ER and Golgi (Kurokawa and Nakano, 2019). MOG1 facilitates the export of Na_V_1.5 from the ER and improves Na_V_1.5 expression on the cell surface, probably by interacting with Sar1A and Sar1B (Chakrabarti et al., 2013; Wang et al., 2018; Yu et al., 2018; Kurokawa and Nakano, 2019). It is now recognized that ER retention signals can also play an important role in ER export of many plasma membrane proteins. After masking the ER retention signals, the proteins seem to receive an “exit card” and are released from the ER. The DI-II linker region of Na_V_1.5 contains three putative RXR-type ER retention motifs (479RKR481, 533RRR535, and 659RQR661). Protein kinase A (PKA)-mediated phosphorylation of Na_V_1.5 masks the retention signals in the DI-II linker region of Na_V_1.5, and promotes the export of Na_V_1.5 from the ER to the Golgi (Zhou et al., 2002; Rook et al., 2012).

Nevertheless, the ER export mechanism of Na_V_1.5 is still poorly understood. Rab GTPases, the largest members of the Ras superfamily, play an important role in protein trafficking between intracellular compartments in eukaryotes (Li and Marlin, 2018). Rab1 and Rab2 are reported to regulate protein transport between the ER and Golgi, but their role in Na_V_1.5 trafficking is uncertain and needs to be tested in further studies.

### Golgi Export

Selective Golgi export of proteins from the Golgi to the plasma membrane is another key step in protein trafficking, which has been studied in inward-rectifying potassium channels (Li et al., 2016). However, little is known about the regulation of Na_V_1.5 transport from the Golgi. It is speculated that Golgi retention and export motifs possibly modulate Na_V_1.5 exit from the Golgi (Gao et al., 2014).

### Anchoring

Cardiac Na_V_1.5 channels are arranged in clusters, also called pools, in the following membrane microdomains of cardiomyocytes: intercalated discs (ID), T-tubules, costameres, and caveolae. ID mainly consist of three intercellular adhesion structures namely, gap junctions, adherens junctions, and desmosomes (Clark et al., 2002). ID is responsible for electromechanical coupling. The upstroke of AP caused by Na^+^ influx through Na_V_1.5 channels can spread quickly through IDs to ensure the coordinated excitation and contraction among cardiomyocytes. T-tubules are tubular invaginations of the lateral sarcolemma that link the sarcoplasmic reticulum and initiate excitation-contraction coupling. AP mediated by Na_V_1.5 is transmitted along the T-tubules, induces calcium release, and further triggers the contraction of cardiomyocytes. Costameres are sarcolemmal transverse rib-like structures that adhere adjacent cardiomyocytes to the extracellular matrix and maintain the three-dimensional structure of the myocardium (Balse and Eichel, 2018). Caveolae are 50–100 nm cave-like invaginations of the plasma membrane that are made up of essential scaffolding proteins called caveolins. Besides sodium channels (Na_V_1.5), L-type calcium channels, potassium channels (K_V_1.5), sodium-calcium exchanger, and Na^+^/K^+^ ATPase have been subcellularly localized to caveolae and may allow these channels to be integrated into macromolecular complexes to exert their functions and to be regulated in a unified manner (Balijepalli and Kamp, 2008). T-tubules, costameres, and caveolae are all located in the lateral membranes of cardiomyocytes. Na_V_1.5 channels are located in different membrane microdomains of cardiomyocytes, which are determined by their specific partners, such as cytoskeleton-binding proteins, gap junctional proteins, and desmosomal proteins (Table 2; Balse and Eichel, 2018).

**TABLE 2:** Protein partners associated with Na_V_1.5 localization in the cardiomyocytes.

Classification	Representation	Localization	References
Cytoskeleton-binding proteins	ankyrin-G	intercalated disks	Makara et al., 2014; Yang et al., 2020
Gap junctional proteins	Connexin 43	intercalated disks	Bruce et al., 2008; Sottas et al., 2018
	Plakophilin 2	intercalated disks	Sato et al., 2009
Desmosomal proteins	Desmoglein 2	intercalated disks	Rizzo et al., 2012
	Desmoplakin	intercalated disks	Zhang et al., 2013
Dystrophin–syntrophin complex	Dystrophin	lateral membranes	Gavillet et al., 2006
Caveolins	Caveolin-3	Caveolae	Cheng et al., 2013; Gavillet et al., 2006; Schilling et al., 2016; Vaidyanathan et al., 2018
MAGUK proteins	SAP97	intercalated disks	Petitprez et al., 2011
	CASK	lateral membranes	Eichel et al., 2016

To our knowledge, a sorting process at the early stages possibly exists, which affects the final anchoring of membrane proteins toward specific microdomains. To better understand the mechanism of Na_V_1.5 anchoring, it is of great value to investigate the complex processes of sorting, identification by protein chaperones, and organization of microdomains, both in physiological and pathological conditions.

## Degradation

**FIGURE 3:**
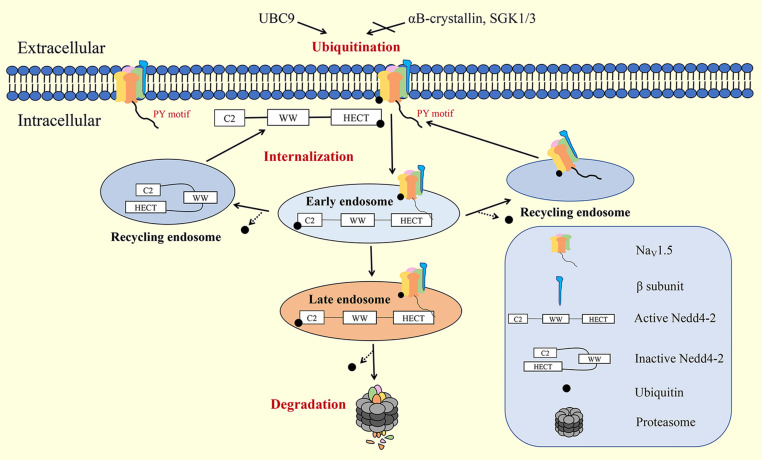
Internalization and degradation of Na_V_1.5 on the plasma membrane. Nedd4-2, neural precursor cell-expressed developmentally downregulated 4 type 2; SGK, serum- and glucocorticoid-inducible kinase.

The last step of the Na_V_1.5 channel life cycle is degradation, which can be considered as a form of retrograde trafficking (Figure 3). There are two major pathways for the degradation of Na_V_1.5: the proteasome and autophagic degradation pathways.

### Proteasome Degradation Pathway

Ubiquitination is a common signal for protein internalization and degradation. E3 ubiquitin ligase Nedd4-2 contains WW domains, an N-terminal calcium/protein binding C2 domain, and a C-terminal HECT domain. When the C2 domain binds to the HECT domain, Nedd4-2 presents an inactive state. When Ca^2+^ binds to the C2 domain, the HECT domain is exposed and Nedd4-2 is activated. Activated Nedd4-2 binds to the PY motifs of target proteins through its WW domains for ubiquitination (Lamothe and Zhang, 2016). Ion channels tagged with ubiquitin are usually compartmentalized into vesicles destined for degradation (by the lysosomal or proteasomal system) or recycled back to the membrane via small GTPase Rabs. Nedd4-2 binds to the PY motif of Na_V_1.5 through WW domains for ubiquitination, which leads to the rapid internalization and degradation of Na_V_1.5 (van Bemmelen et al., 2004). Calcium-dependent Nedd4-2 upregulation accelerates the degradation of Na_V_1.5 and decreases the *I*_Nac_ density on the plasma membrane (Luo et al., 2017). αB-crystallin (Huang et al., 2016), serum- and glucocorticoid-inducible kinase (SGK) (Boehmer et al., 2003), and UBC9 (Tang et al., 2019) are reported to interact with Nedd4-2 to regulate the degradation of Na_V_1.5.

### Autophagic Degradation Pathway

Adenosine 5′-monophosphate-activated protein kinase (AMPK) plays a major role in mediating the autophagic degradation of Na_V_1.5 during cardiac ischemia and reperfusion (I/R) injury. I/R stimulation was reported to downregulate Na_V_1.5 abundance in rat hearts. An *in vitro* study showed that under conditions of I/R stress, Na_V_1.5 was degraded through the AMPK-mediated autophagic pathway rather than through the proteasome pathway (Tang et al., 2019). AMPK assists Na_V_1.5 to bind to the autophagic adapter protein and microtubule-associated protein 1 light chain 3 (LC3) by phosphorylating Na_V_1.5 at threonine (T) 101, and enhances the subsequent degradation through the autophagic pathway (Liu et al., 2019).

## Modulation of the Gating Properties

Na_V_1.5 activation depends on the S4 segment, which contains multiple positively charged amino acids that move across the membrane in response to changes in membrane potential. The voltage-sensing domain (VSD), formed by S1-S4, rotates when membrane depolarization moves the S4 segment within DI–DIII outward relative to other channel segments. This allows Na^+^ to flow inwardly through the pores composed of the S5 and S6 segments and the P loop. Early *I*_Na_ induces rapid AP upstroke, leading to further channel activation. Na_V_1.5 inactivation involves two distinct processes: fast and slow inactivation. Fast inactivation is coupled to the activation process and occurs within milliseconds. This process may involve an isoleucine-phenylalanine methionine (IFM) motif, which is a highly conserved amino acid triplet in the III–IV linker (Chadda et al., 2017). Unlike fast inactivation, slow inactivation is not coupled to the activation process and can last for as long as a few seconds. Conformational modifications in the following three domains drive the slow inactivation process: the trans-membrane segment S4/D4, the loop between S5-S6/DII (P-loop/DII), and segment S6/DII (Detta et al., 2015). When Na_V_1.5 channels do not completely inactivate, a lasting current less than 0.5% of the peak current can be detected in the cardiomyocytes, which is known as late *I*_Na_ (*I*_Na–L_). Because of the short duration of the AP plateau, *I*_Na–L_ can result in situations where the Na^+^ “load” becomes twice as much as peak *I*_Na–P_ over time, and this plays an important role in angina, arrhythmia, and HF (Makielski, 2016). Many proteins have been identified to modulate the gating properties of Na_V_1.5, such as β-subunits, FGF, CaMKII, and 14-3-3. Their interacting domains and modulation effects are summarized in Table 3.

**TABLE 3:** Proteins and their modulation effects on the gating properties of Na_V_1.5 channels.

Proteins		Interacting domains	Modulation effects of gating properties	References
β-subunits	β1	DIV-VSD	Co-expression of β1 with cardiac Na_V_1.5 in Xenopus oocytes increased *I*_Na_ amplitude, resulting in a depolarizing shift in steady-state inactivation and enhances recovery from inactivation. Overexpressing β1 caused significant hyperpolarizing shifts in both activation and inactivation kinetics, and greatly reduced persistent *I*_Na–L_ in the Chinese hamster ovary cells stably expressing human Na_V_1.5.	Fahmi et al., 2001; Ko et al., 2005; Zhu et al., 2017
	β2		Co-expression of β2 with Na_V_1.5 caused a negative shift in activation in Pro5 and Lec2 cells. Co-expression of β2 with Na_V_1.5 caused a positive shift in inactivation in Chinese hamster ovary cells.	Johnson and Bennett, 2006; Watanabe et al., 2009
	β3	DIII-VSD	Overexpressing β3 accelerated macroscopic current decay, caused hyperpolarizing shifts in both activation and inactivation kinetics, slowed recovery from inactivation, and greatly reduced persistent *I*_Na–L_ in the Chinese hamster ovary cells stably expressing human Na_V_1.5.	Ko et al., 2005; Zhu et al., 2017
	β4		Controversial. Medeiros-Domingo’s study showed that overexpression of β4 with Na_V_1.5 significantly increased the slope factors of both activation and inactivation, shifted the inactivation curve to a more negative potential, and reduced slow recovery from inactivation in HEK293 cells stably expressing Na_V_1.5. Tan’s study showed that co-expression of β4 with Na_V_1.5 did not affect the activation or recovery from inactivation of the cardiac sodium current, but significantly shifted the inactivation curve to a more negative potential in HEK293 cells. However, in Qin Yang’s study, they did not observe any significant effect.	Medeiros-Domingo et al., 2007; Tan et al., 2010; Yang et al., 2019
FGF	FGF12	C-terminus	Overexpression of FGF12 exhibited a significant hyperpolarizing shift in voltage-dependent inactivation without affecting the activation of Na_V_1.5.	Liu et al., 2003
	FGF13	C-terminus	The knockout of FGF13 in adult mouse ventricular myocytes delayed recovery from inactivation of Na_V_1.5.	Wang et al., 2011
CaMKII		IQ motif	Overexpressing CaMKII exhibited a depolarizing shift in sodium channel inactivation and increased *I*_Na–L_ in rabbit, mouse and the guinea pig ventricular cardiomyocytes. Its effects on recovery from inactivation and intermediate or slow inactivation of Na_V_1.5 are controversial.	Aiba et al., 2010; Wagner et al., 2006
14-3-3η		the first interdomain of Na_V_1.5 between amino acids 417 and 467	The co-expression of Na_V_1.5, β1 subunit and 14-3-3η in the COS-7 cells resulted in a negative shift in inactivation and a delayed recovery from inactivation without changes in the activation and current density. 14-3-3η mediated coupled gating.	Allouis et al., 2006; Clatot et al., 2017

## Post-Transcriptional Modifications

Interacting proteins modify the structure and properties of Na_V_1.5 through post-transcriptional modifications (PTMs), such as glycosylation, phosphorylation, methylation, acetylation, and ubiquitylation. PTMs, such as PKA- and CaMKII-mediated phosphorylation and Nedd4-2-mediated ubiquitylation have been discussed in the former sections. We will put more emphasis on the glycosylation, SUMOylation, methylation, and acetylation of Na_V_1.5 in the following sections.

### Glycosylation

Many ion channels are known to contain glycan moieties oriented toward the outside of the membrane, which are anchored to residues, such as serine, threonine (O-linked glycosylation), or asparagine (N-linked glycosylation) (Marionneau and Abriel, 2015). Sialic acids, which are negatively charged terminal residues, are subsequently linked with N-glycans or O-glycans through trans-Golgi sialyltransferase activity and contribute to the extracellular surface potential. The Na_V_ is a heavily N-glycosylated protein with carbohydrates that make up 20 to 30% of total mass. However, Na_V_1.5 is an exception, with approximately 5% by weight of carbohydrates (Mercier et al., 2015). N-glycosylation, which is initiated in the ER and terminated in the Golgi apparatus, is reported to be essential for membrane localization and gating properties. The D25N mutation of the β1 subunit resulted in decreased targeting of Na_V_1.5 to the plasma membrane due to a maturation (glycosylation) defect (Baroni et al., 2018). Ventricular Na_V_ from ST3Gal4 (a glycogene responsible for glycoprotein sialylation) deficient mice is reported to exhibit gating at higher depolarized potentials, slower inactivation, and faster recovery than in control mice (Ednie et al., 2013).

### Methylation

Arginine methylation within the interdomain linker between DI and DII of Na_V_1.5 is catalyzed by protein arginine methyltransferases (PRMT), which transfer methyl groups from the cofactor S-adenosyl-L-methionine (SAM) to the protein. Arginine methylation is an important PTM in voltage-gated ion channels. PRMT-3 and -5 methylate Na_V_1.5 and increase Na_V_1.5 current density by increasing the cell surface expression in HEK293 cells (Beltran-Alvarez et al., 2013). Arginine methylation of Na_V_1.5 is also detected in the human heart tissue of patients with end-stage HF. Additionally, R526 methylation is identified as the major Na_V_1.5 methylation site in patients with end-stage HF (Beltran-Alvarez et al., 2014). Moreover, the methylation-phosphorylation crosstalk of Na_V_1.5 has been previously reported. Na_V_1.5 methylation at R513 significantly decreased S516 phosphorylation, which consequently hindered R513 methylation (Beltran-Alvarez et al., 2015).

### Acetylation

N-terminal acetylation, which is irreversibly catalyzed by ribosome-associated N-terminal acetyltransferases (NATs), is a major PTM of eukaryotic proteins that regulates protein–protein interactions and membrane targeting. Recently, N-terminal acetylation has been reported to be a degradation signal that inhibits protein targeting to the secretory pathway (Arnesen, 2011). N-terminal acetylation of Na_V_1.5 at the initial 30 alanine residues was discovered in the hearts of patients with end-stage HF (Beltran-Alvarez et al., 2014). In contrast, N-lysine acetylation, which is catalyzed by histone acetyltransferases (HATs), can be reversed by histone deacetylases and sirtuins. Sirtuin1 deacetylase-mediated acetylation at lysine 1479 of Na_V_1.5 results in enhanced trafficking to the surface of the plasma membrane and increased *I*_Na_ (Yoon et al., 2017).

### SUMOylation

SUMOylation, a PTM, is analogous to ubiquitylation involving an enzymatic cascade. In contrast to ubiquitylation, SUMOylation involves conjugating the proteins to small ubiquitin-like modifiers (SUMOs). SUMOylation can affect protein structure and subcellular localization. Recently, Na_V_1.5 was reported to be SUMOylated only at lysine 442. The mutation of lysine 442 or application of a deSUMOylating enzyme inhibits hypoxic reopening in response to hypoxia. SUMOylation of Na_V_1.5 is associated with an increase in *I*_Na–L_ (Plant et al., 2020).

Recently, novel post-transcriptional modifications have been reported. Zhang et al. found that an endogenous “lactate clock” turns on gene expression to promote homeostasis in bacterially challenged M1 macrophages (Zhang et al., 2019). The presence of histone lactylation represents a new avenue for understanding the pathophysiological mechanism of many diseases, including ischemic cardiovascular disease. It has been established that Na_V_1.5 channels are significantly downregulated under conditions of ischemia and hypoxia, which is accompanied by the accumulation of lactic acid. Whether lactylation is involved in the regulation of Na_V_1.5 under pathological conditions has not yet been revealed.

## *SCN5A*-Related Diseases and Potential Therapy Strategy

Na_V_1.5 is associated with the pathogenesis of various cardiovascular diseases, such as arrhythmia, ischemic cardiomyopathy, heart failure, and sudden cardiac death. Most cases of cardiac arrhythmia and sudden cardiac death are caused by *SCN5A*-related mutations, which change Na_V_1.5 expression and function. The gain-of-function mutations in *SCN5A*, which lead to increased *I*_Na–L_, can lead to long QT syndrome 3 (LQT3). Loss-of-function mutations in *SCN5A*, which lead to decreased *I*_Na_, can cause Brugada syndrome (BrS), sick sinus node syndrome, and atrial fibrillation (Li et al., 2018). To date, the reported BrS-associated *SCN5A* mutations and LQT3-associated mutations are over 300 and over 80, respectively. Previous studies have demonstrated that Na_V_1.5 expression is downregulated in HF (Luo et al., 2017). The upregulated expression of Nedd4-2, co-localization of Nedd4-2 with Na_V_1.5, and increased diastolic calcium concentrations were observed in the volume-overloaded HF rat model, suggesting that Nedd4-2-mediated ubiquitination plays an important role in the downregulation of Na_V_1.5 in HF (Luo et al., 2017). *SCN5A* variants are now recognized to be involved in the pathogenesis of HF. A1180V, an *SCN5A* variant, decreased *I*_Na_ and moderately increased *I*_Na–L_. A1180V carriers without cardiac dysfunction initially exhibit deteriorated cardiac functions and progress to HF or atrioventricular block during follow-up (Ge et al., 2008). Although some studies have elucidated the correlation between Na_V_1.5 and HF, the specific mechanisms are not yet known.

Considering the important role of Na_V_1.5 in these diseases, novel therapeutic strategies targeting the biosynthesis, trafficking, and sodium current of Na_V_1.5 have been developed.

### Treatment Targeting Biosynthesis of Na_V_1.5

Several studies have proposed that promoting the process of translational readthrough can enable ribosomes to neglect the stop codon to produce full-length proteins and reduce the harmful results caused by nonsense mutations to a certain degree. Pharmacological strategies to enhance translational readthrough include aminoglycosides, small-interfering RNA (siRNA) that target eukaryotic release factors (eRF), and suppressor tRNAs that recognize stop codons (Carnes et al., 2003). This idea was tested in the nonsense mutation *SCN5A*-W822X by using aminoglycosides and siRNA against eRF3a and proved effective, which might be a novel treatment strategy for nonsense *SCN5A* mutation carriers (Teng et al., 2009). However, its application in clinical practice is largely limited due to safety concerns, such as introducing sequence changes to restored full-length proteins.

### Treatment Targeting Trafficking of Na_V_1.5

H558R, a common *SCN5A* polymorphism, restores the defective trafficking of BrS-associated *SCN5A* mutations R282H, S216L, K317N, and D1690N by promoting proper protein folding (Marangoni et al., 2011; Shinlapawittayatorn et al., 2011; Nunez et al., 2013). The mechanisms by which H558R rescues *SCN5A* mutations are controversial. Decreased *SCN5A* promoter methylation by H588R is one of the mechanisms involved (Matsumura et al., 2017). In an *in vitro* study, small peptides that span the H558R polymorphism, have been shown to be sufficient in restoring the trafficking defect of BrS-associated *SCN5A* mutations-R282H, indicating that it might be possible to use R558-containing peptides as a novel strategy tailored to specific BrS mutants (Shinlapawittayatorn et al., 2011). However, the rescue effects *in vivo* still need to be investigated.

### Treatment Targeting *I*_Na–L_ of Na_V_1.5

Ranolazine, eleclazine, amiodarone, mexiletine, flecainide, and quinidine targeting *I*_Na–L_ have been widely studied for the treatment of LQT3 patients (Makielski, 2016). For example, we take mexiletine and ranolazine. Ranolazine, which is approved for the treatment of angina pectoris, preferentially reduces *I*_Na–L_. To date, Ranolazine has been investigated with respect to *SCN5A* mutations such as ΔKPQ, Y1767C, R1623Q, and D1790G (Moss et al., 2008; Rajamani et al., 2009; Huang et al., 2011; Chorin et al., 2016). Mexiletine, an oral class Ib anti-arrhythmic agent, targets cardiac Na_V_1.5, and preferentially inhibits *I*_Na–L_. It has been reported that mexiletine can effectively shorten the QT interval in a subset of patients with LQT3, while other patients are insensitive to the drug (Mazzanti et al., 2016). The conformation of the less activated DIII voltage-sensing domain (DIII-VSD) of Na_V_1.5 is hypothesized to be the cause for mexiletine insensitivity in these patients (Zhu et al., 2019). A combination of mexiletine with a drug that can promote DIII-VSD activation can be a novel therapeutic strategy for these mexiletine-insensitive patients.

## Conclusion and Perspectives

The life cycle of Na_V_1.5 begins with the transcription and translation of the *SCN5A* gene. Na_V_1.5 is then trafficked from the ER to the Golgi apparatus and subsequently to the sarcolemma and cytoskeleton. Finally, Na_V_1.5 is internalized and degraded. In the past decades, great achievements have been made in the regulation of Na_V_1.5 from biosynthesis and trafficking to degradation. The knowledge we have generated helps in the understanding of the pathogenesis of *SCN5A*-related diseases and management of these patients. However, the complete life cycle of Na_V_1.5 has not been fully mapped. In the end of each section, we have pointed out the corresponding unsolved problems. All these problems can be summarized in two sections: regulation of Na_V_1.5 under physiological conditions and in pathological conditions. We believe that with joint efforts, we will achieve a deeper understanding of the life cycle of Na_V_1.5 and explore more effective treatment strategies to reduce mortality and improve the quality of life of patients suffering from *SCN5A*-related diseases.

## Author Contributions

CD and YW drafted the manuscript. AM and TW conceived and supervised the review. All authors contributed to the article and approved the submitted version.

## Conflict of Interest

The authors declare that the research was conducted in the absence of any commercial or financial relationships that could be construed as a potential conflict of interest.
